# Parity-time and anti-parity-time symmetries in heat transfer

**DOI:** 10.1093/nsr/nwae275

**Published:** 2024-08-10

**Authors:** Jingwen Ma, Xiang Zhang, Xiaobo Yin

**Affiliations:** Department of Physics, The University of Hong Kong, China; Department of Physics, The University of Hong Kong, China; Department of Electrical and Electronic Engineering, The University of Hong Kong, China; Department of Mechanical Engineering, The University of Hong Kong, China; Department of Physics, The University of Hong Kong, China; Department of Mechanical Engineering, The University of Hong Kong, China

## Abstract

This perspective briefly reviews the recent developments of non-Hermitian parity-time and anti-parity-time physics in dissipative heat transfer systems, highlighting their potentials in novel functional thermal devices in the future.

The concepts of parity-time (PT) symmetry [1] and its conjugate, anti-PT (APT) symmetry [2], have led to opportunities for studying new and captivating wave dynamics. Incorporating non-Hermitian potentials, such as fine-tailored gain/loss doping profiles and dissipative coupling, allows the establishment and observation of PT and APT symmetries in wave propagation [3]. Systems exhibiting PT/APT symmetries enable real eigen spectra and undergo a transition at a specific point in parameter space in which eigenvalues and eigenstates coalesce simultaneously. This point, known as the exceptional point (EP), is associated with a wide range of intriguing physical phenomena, including single-mode lasing [4], unidirectional invisibility observed in optics/acoustics [5] and robust wireless power transfer [6]. In stark contrast to wave systems, macroscopic heat transfer follows Fourier's law and possesses inherent non-Hermitian characteristics due to its dissipative nature. This makes heat transfer an advantageous system for exploring non-Hermitian physics, as it allows direct manipulation of non-Hermitian potentials through diffusivity modulation.

In 2019, Li *et al.* proposed a method for achieving APT symmetry in heat transfer (left panel in Fig. 1a). This was realized by introducing wave-like fluctuations driven by opposite convections in two counter-rotating rings (Fig. 1b) [7]. The temperature field exhibited a wave-like solution, with the modulated amplitude defined as the difference between the maximum temperature (*T*_max_) and the reference temperature (*T*_ref_), and the thermal phase ($\varphi $) was represented by the position of *T*_max_. The eigenfrequencies of such a system are $\omega = - i[ {( {{{k}^2}D + {{h}_0}} ) \pm \sqrt {h_0^2 - {{k}^2}{{v}^2}} } ]$, whose real and imaginary parts describe the phase velocity and effective diffusivity, respectively. Here, *k* is the effective wave number, *D* is the diffusivity, $\pm v$ are the rotational velocities of the two rings and ${{h}_0}$ is the rate of heat exchange between them. When the rotational velocity *v* is below the threshold ($v = {{h}_0}/k$), the system exhibits APT symmetry and has purely imaginary eigenfrequencies (${\mathrm{Im}}\ ( \omega )$, representing effective diffusivity). In this regime, the temperature profiles show dynamic localization with a static phase lag. When the rotational velocity *v* is increased, the imaginary part of the eigenfrequencies gradually merges at the EP (Fig. 1c). Remarkably, for rotational velocity *v* that is above the EP threshold, the system enters an APT symmetry broken regime and its nonzero real eigenfrequencies (${\mathrm{Re}}\ ( \omega )$, representing phase velocity) lead to a wave-like temperature profile that is continuously moving with the rotational background.

**Figure 1.**
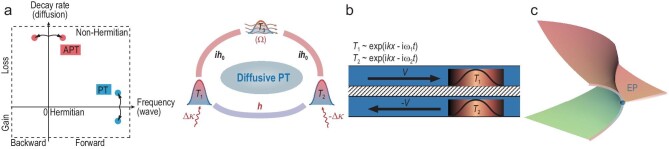
Parity-time (PT) and anti-PT (APT) symmetries in heat transfer. (a) Establishment of APT (left panel) and PT (right panel) symmetries in heat transfer. (b) Wave-like fluctuations are driven by directional convections. (c) Eigenvalues coalesce at the EP and the system undergoes a transition from the PT/APT symmetry regime to the PT/APT symmetry broken regime. Adapted with permission from Refs [7,10].

The discovery of APT symmetry in heat transfer has ushered non-Hermitian physics into diffusive systems, enriching the understanding of temperature field evolution by considering wave-like Hermiticity and physical symmetry [8]. The modulation of thermal phases through convection has introduced a new means of thermal regulation: the effective Hamiltonian method inspired the exploration of thermal topology from the bandgap design perspective of heat transfer structures [9]. However, achieving PT symmetry in heat transfer remains theoretically and experimentally challenging because real coupling in thermal diffusion systems is counterintuitive due to the inherently dissipative nature of heat transfer, and the negative thermal conductivity required by the exact PT symmetry is also unattainable.

Recently, Cao *et al*. proposed a mechanism of real coupling in heat transfer and resolved the nontrivial puzzle of diffusive PT symmetry [10]. As depicted in the right panel of Fig. 1a, a real coupling between the two temperature fields (*T*_1,2_) can be effectively established by using an intermediate strong convection background ($\Omega = kv,{\mathrm{\ }}$*T*_3_). Since $\Omega $ was sufficiently strong and *T*_3_ quickly faded into the steady state, the original dissipative coupling ($i{{h}_0}$) between adjacent temperature fields (*T*_1_ and *T*_3_, *T*_2_ and *T*_3_) was transformed into an effective real coupling ($h = \frac{{h_0^2}}{\Omega }$) between *T*_1_ and *T*_2_. By introducing thermal conductivity detuning (anisotropic design in the experiment), they constructed diffusive PT symmetry and predicted the transition at EP. The amplitude and phase of temperature fields were precisely controlled, and the transition from PT symmetry to the PT symmetry breaking phase was observed. As ${{h}_0} \ll \Omega $, the phase oscillation induced by real coupling was sufficiently suppressed in both the PT symmetry and PT symmetry broken regimes.

PT symmetry in heat transfer enriches our understanding of energy exchange and enhances our current comprehension of thermal diffusion by uncovering a new physical symmetry in the diffusive system. The realization of real coupling provides a general approach to exploring diverse wave-like effects in heat transfers, allowing the effective suppression of thermal phase oscillation and precise thermal regulation.

The exploration of PT and APT symmetries in heat transfer offers promising opportunities for precise thermal management based on symmetry considerations. Heat transfer serves as a natural platform to explore non-Hermitian physics and wave-like phenomena in diffusive systems, drawing parallels with wave dynamics. Unlike previous realizations of PT and APT symmetries in wave systems, which required artificially engineered non-Hermitian potentials, heat transfer systems provide a unique platform that is naturally non-Hermitian and inherently dissipative. The non-Hermitian properties of heat transfer systems arise from the fundamental characteristics of the system, rather than being the results of external modulations. Notably, the emerging non-Hermitian phenomena could be observable in heat transfer systems even in the absence of thermal convection. We anticipate that innovative thermal phase regulation based on diffusive PT symmetry will inspire the development of novel functional thermo-devices, such as precise sensors and robust thermal collectors.
